# No wrong door: addressing injustices and achieving better mental healthcare provision for under-18s in acute physical healthcare settings

**DOI:** 10.1192/bjb.2021.1

**Published:** 2022-02

**Authors:** Virginia Davies

**Affiliations:** Whittington Hospital, London, UK

**Keywords:** Paediatric mental health, commissioning, inequity, childhood experience, comorbidity

## Abstract

The distressing reality that mental healthcare for children and young people in acute trust settings in the UK is woefully underprovided is not news. But with acute trust debts being written off, hospital trusts and commissioners of services have a timely opportunity to address this age- and condition-based discrimination.

Delivering a just service for under-18s depends on attitude, resources and adequate knowledge of the tasks involved. This article aims to describe the current landscape, summarise the arguments for better integrating mental healthcare into physical healthcare settings, articulate the tasks involved and the challenges for commissioning and providing, and finally share examples of current service models across the country.

Ultimately, commissioning and provider choices will be constrained by resource pressures, but this article aims to underscore why commissioning and providing a portmanteau ‘no wrong door’ hospital service for children, young people and families is worth the headache of thinking outside old commissioning and provider boxes.

In 2019, the National Confidential Enquiry into Patient Outcome and Death (NCEPOD) report into the mental healthcare of young people in the UK^1^ concluded that:

- mental healthcare was not given the same level of importance as physical healthcare in general hospitals
- general hospital staff were not receiving enough support from mental health professionals in the general hospital setting, particularly with regard to risk management.

Despite these damning findings, the report did not advise commissioners how they could use their purchasing power to exact a more equitable provision of mental healthcare for young people in hospital settings. Unhelpfully, in terms of systems change, many of NCEPOD's recommendations can be implemented at a ‘tick box’ level, through superficial changes to job definitions and training plans.

And, having stated in 2015 that ‘What is particularly worrying is that children with physical, learning or mental health needs are telling us they have poorer experiences [in hospitals]’,^2^ Ted Baker, the Care Quality Commission's chief inspector of hospitals, noted in the 2020 Assessment of Mental Health Services in Acute Trusts (AMSAT) report^3^ that:

‘Physical and mental health care have traditionally been delivered separately. While investment and improvements in mental health services are welcome, physical and mental health services will only truly be equal when we stop viewing physical and mental health as distinct. Services need to be built around all of people's needs and not determined by professional or interest groups.’

He continues:

‘Many of the people attending acute hospital emergency departments with physical health needs may also have mental health needs. These people are in a vulnerable position and need to be treated with compassion and dignity. This must be in a way that makes them feel safe and upholds their human rights. In our report, we raise concerns that people with mental health needs are not always receiving this level of care. How well they are treated in an emergency department, or elsewhere in an acute hospital, is often linked to the importance that mental healthcare is given by the trust board. Acute trusts must do more, but they also need support from mental health trusts to develop better and more integrated approaches to care.’

AMSAT makes some welcome recommendations for integrated care systems and acute trusts; however, with no absolute commissioning directives regarding ‘whole person’ hospital care, most trusts will choose to overlook this central aspect of patients’ – and especially children's – care.

The tendency for adult and physical health priorities to set the agenda within acute trusts means that children and young people with mental health needs seem always to be last in the queue. This is despite the well-known rates of comorbidity between long-term physical and mental health conditions in children (Fig. 1)^4–7^ and the immediate, let alone long-term, resource implications of failing to address these psychiatric comorbidities.^8^

**Fig. 1:**
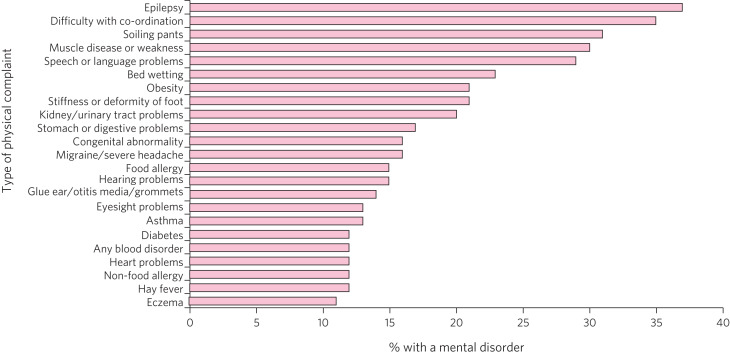
Prevalence of mental disorders in children with specific physical complaints. From Meltzer et al, p. 74.^4^ © Crown copyright 2000, see http://www.nationalarchives.gov.uk/doc/open-government-licence/version/3/.

## The levers of integration

As AMSAT points out, if integrated treatment of mind and body is to be achieved, it must be underpinned by effective service-level agreements between stakeholders. The principles that guide such contracts were well articulated in *Side by Side*,^9^ published in February 2020. This UK-wide consensus statement, agreed by the Royal Colleges of Psychiatrists, Nursing, Emergency Medicine and Physicians, calls on all parties to work together to better care for patients with mental health needs in acute hospitals.

‘Best care’ is characterised by:

- reciprocal competencies in each staff group, physical and mental
- joint ownership of the care of children and young people while in the hospital
- co-location of physical and mental health staff.

Addressing the second and third aspects, reciprocal competencies and joint ownership of care, can be relatively easy, but as Ted Baker observed in AMSAT: ‘Where high-quality leadership for better mental health in acute trusts was lacking, we saw how there was more likely also to be a lack of appropriate training to support staff and poor working relationships between acute and mental health trusts.’^3^

### Reciprocal competencies

Exchange programmes for junior doctors and nurses are already in place in some areas. Likewise, many health practitioner training programmes now contain modules offering reciprocal competency qualification, and frameworks such as the UCL competency framework^10^ allow staff to register as having reached various competencies in relation to mental health training. This model could be used to determine levels of mental health competency and capacity within the acute trust workforce. Those aiming to improve capacity in this area should be aware that the ‘We Can Talk’ training^11^ used by many trusts to help staff to feel better equipped to talk about mental health problems with children and young people, detect safeguarding issues and provide signposting is not a child and adolescent mental health services (CAMHS) competency framework. Consequently, adoption of this training across a trust should not be used to distract from inadequate mental health staffing. Both are needed: upskilling of physical health staff, as well as direct employment of specialist mental health staff.

### Joint ownership

Joint ownership of patient care can be interrogated by examining a trust's pathways and protocols. These agreements can usefully confirm which team will take lead responsibility for a young person's care. Children and young people who have used hospital emergency departments during mental health crisis describe how the experience of feeling unwanted at a time of particular vulnerability puts them off returning.^12^ Given increasing rates of self-harm and suicide in young people,^13,14^ this is not a desirable outcome.

### Co-location of staff

The biggest challenge to achieving genuine side-by-side working is co-location of physical and mental health staff. This is not simply a problem of estate management and a lack of space – it is because co-location of mental and physical healthcare provision presents a challenge to the very notion of what an acute hospital is about. Acute trust functioning and the commissioning of services within hospitals remains mired in an outdated notion of physical healthcare. Within this conceptualisation, physical health is divorced from the unconscious and from emotional and irrational reactions to physical ill health and disease, let alone family psychological factors, and care packages are linear processes.

## Key considerations in commissioning integrated care for children and young people

Four main areas need to be considered when negotiating contracts for integrated acute trust care for under-18s:

- the range and complexity of mental health tasks to be addressed
- commissioning discontinuities and fragmentation between adult and child, mental and physical, local and regional/national/international services
- funding sources for non-patient-facing activities, including staff support and professional development
- ensuring a single ‘front door’ for children and young people and their families.

### Tasks to be addressed

Broadly speaking, three mental health tasks need to be managed in the acute trust setting: crisis/emergency mental health presentations; non-urgent psychiatric or psychological problems; systems issues regarding complex cases. Box 1 gives more detail.

Box 1 Mental health tasks relating to under-18s to be managed in the acute trust setting

*Crisis/emergency mental health presentations*. These presentations involve under-18s in the emergency department or on the ward who need urgent joint assessment, alongside physical monitoring with or without treatment. Some individuals may need an emergency place of safety within the hospital. They might include children and young people with self-harm and attempted suicide, psychosis, acute confusional states (delirium), eating disorders and sudden deterioration in behaviour in the context of autism spectrum disorders or intellectual disability. A significant proportion will have safeguarding needs. Some children and young people will have psychiatric needs related to physical health medicines (e.g. intensive care medicines) or their physical condition (e.g. brain injury).

*Non-urgent psychiatric or psychological problems in in-patients, day patients or out-patients*. This group might include children and young people with medically unexplained physical symptoms such as pain or paralysis, those experiencing major emotional reactions following a newly diagnosed long-term condition, for example non-adherence with medication in asthma or insulin-dependent diabetes mellitus, those with a psychiatric condition in the context of a long-term physical condition, such as attention-deficit hyperactivity disorder in the context of epilepsy, and those subject to medical child abuse/fabricated and induced illness.

*Systems issues regarding complex cases*. Physical health staff dealing with cases involving complicated systems dynamics or complex child or parent psychopathology need access to support, training and consultation from expert mental health colleagues to effectively manage the staff effects that can ensue. These can include conflict within teams (splitting), accidental medical harm of children and young people, inadvertent collusion with abusive parents and staff burnout. Mental health staff embedded with their physical health colleagues can run reflective groups, facilitating psychological processing and providing in-context staff support. Such reflective groups have been shown to reduce staff sickness and burnout in physical healthcare staff.^12,13^

### Commissioning discontinuities and fragmentation

**Fig. 2:**
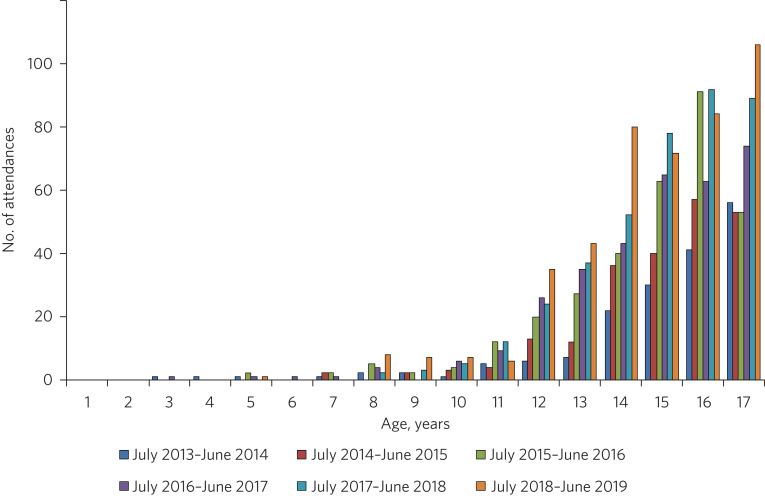
Under-18s requiring emergency mental health assessment in the emergency department of one London teaching hospital over the period 2013–2019.

Commissioning discontinuities and fragmentation are rife for under-18s in hospital, with 16- and 17-year-olds most disadvantaged despite having the highest rates of psychological morbidity (Fig. 2).^15^

The age discontinuity between paediatric commissioning and CAMHS commissioning, especially given the former's non-alignment with educational transition points, is surely an area for urgent attention by integrated care systems (ICSs) (Box 2). ICSs are tasked with breaking down barriers to care as part of delivering the National Health Service's long-term plan,^16^ but with the COVID-19 pandemic having changed the commissioning landscape, how will the new block contracts affect this?

Box 2 Ensuring that 16- and 17-year-olds are not forgotten

Paediatric commissioning finishes at 16, but CAMHS commissioning finishes at 18. The physical arrangement of acute trusts, with most paediatric emergency departments and wards having an age cut-off of the 16th birthday, means that the over-16s end up in environments that are far from young-person friendly. Having no in-house under-18s mental health staff to visit them in these ‘inappropriate’ settings doubly disadvantages the under-18s; their adult equivalents are far more likely to have access to in-house liaison psychiatry teams, since commissioning for adult mental healthcare in hospitals is more advanced than that for under-18s.

How does the commissioning arrangement work when a hospital functions not only as a local ‘district general’, but also as a regional, national and possibly international specialist referral centre? Most acute trusts have arrangements in place for costing physical healthcare packages involving national and international patients, but these rarely take into account potential mental health needs. Greater recognition needs to be given to this side of the ‘business’ and financial packages developed accordingly.

### Funding for non-patient-facing activities

Funding sources for non-patient-facing activities, including staff support, are vital for the sustainability of any integrated service. Significant amounts of non-patient-facing activity are involved in the first two tasks listed in (Box 1): dealing with crisis/emergency mental health presentations and non-urgent psychiatric or psychological problems. A 75-min crisis consultation will often require as much time again, often more, liaising not only with other hospital and primary care staff, but also other agencies, especially social care and education, as well as adult mental health if parental mental illness is a factor. Emergency tariffs rarely cover the hours of work involved or the numbers of mental health staff who may need to be involved. Tariffs need to contain adequate funding for staff with sufficient knowledge of child and adolescent mental health to complete this important liaison work, and payment by results has often meant that provider trusts end up running these services at a loss.

Mental health staff are also important for delivering staff support, something that has become very obvious during the current COVID-19 pandemic. Plenty of evidence exists for the benefits on staff well-being of reflective practice,^17,18^ but this is rarely factored into commissioning agreements between acute providers and commissioners.

### Ensuring a single ‘front door’

Finally, how does the commissioning arrangement ensure that children and young people and their families are not having to visit multiple ‘front doors’ and tell their story multiple times? Having on-site, integrated mental health staff ensures not only that under-18s and their families have an experience of one extended team caring for them, meaning that any mental health professional coming to see them has a good sense of their physical context and is already well-briefed on their possible mental health difficulties, but, perhaps more importantly, that they can access mental healthcare even if they come from a family or culture where attending CAMHS or having mental health problems is difficult to accept or act upon, and where a separate visit to a mental health clinic simply will not happen.^19^ Equally, if the young person's family of origin is chaotic and/or their emotional and behavioural presentations stem from neglect or abuse, the hospital provides a one-stop shop. This offer is unlikely to be the case if commissioning relies on in-reach from local CAMHS.

## Developing a just and best-fit model

Having reflected on how a local hospital service might deliver or not on good care as articulated above, commissioners and providers planning to establish or enhance integrated hospital care for under-18s within the next commissioning cycle might want to consider the following.

- Is/will the team be multidisciplinary (more common in paediatric liaison/children's psychological medicine teams) or unidisciplinary (as in crisis teams or paediatric psychology services)?
- Are/will the team members be employed by the acute trust or by the mental health trust, with honorary contracts with the acute trust? There are pros and cons to each.
- Does/will the funding come via block contracts or activity-based, condition-specific funding streams? The mental health needs of children and young people are often inchoate and less amenable to being fitted into diagnostic boxes or care bundles. Embedded staff, able to respond to the queries of paediatric staff or the sudden call for help with a child's behaviour or family's emotional response, are invariably more useful than staff tied to specific conditions or workstreams.
- Who does/will do the commissioning? Local children's mental health commissioners are responsible for ensuring adequate 24/7 emergency provision, but who will take on responsibility for in-patient, day-patient and out-patient provision? Will this be agreed on a cost-per-case basis with local children's mental health commissioners or will the acute trust agree tariffs with local, regional and national commissioners that include mental health activity? The latter is certainly more sustainable in terms of paediatric mental health service financial viability.
- Does/will the mental health service involve one team or a multitude of different units within the hospital? In some hospitals, the paediatric psychology service functions separately from the paediatric mental health team (which may be called a paediatric liaison team or children's psychological medicine team), and in some hospitals, the paediatric psychologists are not joined in one service, but are simply members of their condition-specific paediatric teams.

## Examples of some current models for under-18s mental health provision

With these considerations in mind, commissioners and providers can examine which of the following models is best for their acute trust/s. Services at these example trusts are further outlined in the Appendix.

- An acute trust-employed under-18s mental health service covering the emergency department, wards and out-patients. The team delivers in-house training, staff support and reflective practice. This model is followed at the Whittington Hospital, London.
- A mental health trust-employed emergency department psychiatric service (adult practitioners) and CAMHS crisis team which sees under-18s emergency department presentations and those admitted for less than 24 h. An acute trust-employed paediatric (i.e. under-16s) mental health team sees all other cases, including crisis admissions of more than 24 h. A paediatric mental health team delivers in-house training, staff support and reflective practice. This is the model at the John Radcliffe Hospital, Oxford.
- A mental health trust-employed emergency department service, with an on-site under-18s mental health team during normal working hours. An on-site mental health team sees certain groups of in-patients and out-patients as part of acute trust-funded, condition-specific service level agreements (e.g. for Tourette syndrome), as well as ‘generic’ in-patients and out-patients if funding is agreed on a cost-per-case basis by local commissioners. There is a large acute trust-employed, condition-specific paediatric psychology service, separate from the mental health team. A paediatric psychology service delivers in-house training, staff support and reflective practice. This model is followed at the Evelina Children's Hospital and St Thomas’ Hospital, London.
- An acute trust-employed under-25s out-of-hours mental health emergency team as well as CAMHS in-reach during normal working hours. An acute trust-employed community counselling service providing in-reach or outpatient services for children on wards or out-patients, as well as paediatric staff support. This model is followed at the Blackpool Victoria Hospital, Blackpool.

## A binary choice?

In effect, commissioners and providers working within integrated care systems have two broad choices when they consider mental health provision for children, young people and families in acute trust settings:

- an embedded, multidisciplinary children's psychological medicine team, staffed by practitioners such as paediatric psychologists, child and adolescent psychiatrists, child mental health nurses, child psychotherapists, physical therapists and social workers, all directly employed by the acute trust and working across all settings;
- two separate mental health teams, one employed by the mental health trust and seeing crisis/emergencies (uni- or multidisciplinary, with nurses usually providing the unidisciplinary input) and one employed by the acute trust seeing all other patients (uni- or multidisciplinary, with psychologists usually providing the unidisciplinary input).

In an ideal world, where team boundaries are minimised, the first model is preferable. Such embedded services allow children, young people and families access to timely mental healthcare, when and where they need it, with staff versed in their physical health needs and without the long waits that currently plague access to CAMHS. Clinical scenarios involving acute behavioural disturbance on paediatric wards or the need for urgent and ongoing psychiatric care for children and young people in intensive/high-dependency care cannot wait around for funding requests that take weeks to agree. Equally, children and young people with disabling unexplained physical symptoms may not appear to mental health commissioners to be ‘mental’ and legitimate recipients for funding (not fitting usual CAMHS eligibility criteria), so then fall between posts.

It is hoped that this article gives commissioners and providers the questions and framework to query current arrangements and to ask themselves:

- Can children, young people and families in my integrated care system expect a unified care offer when they walk through the front door of our local acute trust/s?
- Will acute trust care costs be contained by having timely mental, as well as physical, healthcare available to the large cohort of under-18 in-patients and out-patients with long-term conditions for whom we are responsible?
- Will under-18s under our care genuinely find that there is no wrong door when they find themselves requiring hospital care?

## About the author

**Virginia Davies**, MRCP, FRCPsych, MRCGP, is a consultant in paediatric liaison in the paediatric mental health team at the Whittington Hospital, London, UK, and chair of the Royal College of Psychiatrists’ Paediatric Liaison Network.

## Appendix

### Detailed service descriptions

#### Blackpool Victoria Hospital, Blackpool

The Child & Adolescent Support & Help Enhanced Response (CASHER) service offers emergency assessment to young people under 25 from 5 pm–10 pm on weekdays and from 10 am–10 pm on weekends and bank holidays. CASHER provides support for young people 365 days a year. CASHER also provide an on-call night time service via their dedicated number (07810 696565) and will come into the hospital to see young people outside of their usual working hours. Each shift is staffed by two mental health staff, one CAMHS-trained and one not. Staff from local CAMHS opt into the staffing rota, which is run by the hospital bank. This avoids any issues with rota absence due to annual leave or sickness. CASHER also offer weekend clinics and drop in sessions for those in crisis. Over 16s are admitted to the adolescent unit or adult medical wards whenever necessary.

CASHER also run an ‘Intensive Home Support’ service (CASHER RAIS) which provides immediate support to young people who may have presented at accident and emergency or are currently on waiting lists for other services. CASHER RAIS ensures that young people are not left unsupported at any stage during their care. CASHER has also adapted their face to face REACH-OUT Groups that are held in the more deprived areas of Blackpool, Fylde & Wyre by supporting online sessions via Zoom with colleagues from Lancashire Children's Services as well as Attend Anywhere for Blackpool Teaching Hospitals online sessions.

CASHER close links to local services including CAMHS and YoutherapY, which are both run by the acute trust. YoutherapY, to which in- and outpatients can be referred or can self-refer, has counsellors working with paediatric staff and children, young people and families in the hospital, as well as working in community sites.

#### Evelina Children's Hospital and St Thomas’ Hospital, London

South London and Maudsley NHS Foundation Trust's National and Specialist Paediatric Liaison Service is a multidisciplinary team focusing on young patients with comorbid medical and psychological conditions (https://www.slam.nhs.uk/national-services/child-and-adolescent-services/paediatric-liaison/).

The team receives referrals from across the UK and internationally for certain conditions and also provides assessment and treatment of in-patients at the Evelina Children's Hospital and St Thomas’ Hospital. The service comprises four consultant psychiatrists, a clinical nurse specialist, a family therapist, a counselling psychologist and specialist training doctors.

Staff are employed by the local mental health trust, with funding coming from a mixture of sources, including portions of the local CAMHS block contract, cost-per-case funding for in- and out-patient work from mental health commissioners and acute hospital funding via service level agreements related to particular conditions, such as tics and Tourette syndrome.

#### Oxford University Hospitals Children’s Psychological Medicine (CPM) service

Oxford University Hospitals Children's Psychological Medicine (CPM) service is primarily staffed by paediatric psychologists, with 2.2 whole-time equivalent child and adolescent psychiatrists. All staff are employed by Oxford University Hospitals NHS Foundation Trust (OUH). OUH has a large adult psychological medicine service, and the child and adolescent psychiatrists are managed within this larger group of adult psychiatrists. OUH's John Radcliffe Hospital is a trauma centre and it receives children who have sustained complex trauma following suicide attempts.

All CAMHS emergencies presenting to the emergency department are seen by the emergency department psychiatry service, which is provided by the local mental health trust. Any children needing in-patient care beyond 24 h, e.g. for medical treatment of an overdose, are then managed by CPM. The adult psychological medicine consultants provide out-of-hours Responsible Clinician cover for all children and young people detained at OUH. The child and adolescent psychiatrists do not undertake any out-of-hours work.

CPM and psychological medicine are funded by out-patient and in-patient tariffs. Some work is funded using best practice tariffs, some by service level agreements with specific teams and some is paid for by monies coming in for medical student teaching. Oxford's Children's Hospital also purchases generic CPM child and adolescent psychiatrist input using money from their overall budget, charged by OUH to commissioners. Any new service development has a small amount immediately factored into the costings to cover CPM or psychological medicine costs.

#### Whittington Hospital, London

Whittington Health NHS Trust's paediatric mental health team (PMHT) at the Whittington Hospital, London, is staffed by psychiatry, nursing, family therapy and psychotherapy (see https://www.whittington.nhs.uk/default.asp?c=25315).

The service offers liaison input to the paediatric team, in-patients and out-patients, and crisis assessments and management in Whittington Hospital's emergency department and the paediatric ward. The service also supports staff on neonatal intensive care.

The PMHT is part of acute paediatrics. The latter is commissioned within the context of the national contract for acute hospital services. Since the PMHT is not a commissioned service, it has to be funded out of the paediatric budget. Whittington paediatrics have been commissioned under Payment by Results for a number of years, with income generated from attendances / admissions. However, this has changed as part of the Covid finance / contracting arrangements and services are now paid for as a block contract. The contract amount is fixed and based on historic expenditure and demand trends.
